# Interferon-γ acutely augments inhibition of neocortical layer 5 pyramidal neurons

**DOI:** 10.1186/s12974-020-1722-y

**Published:** 2020-02-22

**Authors:** Gabriel M. S. Janach, Olivia Reetz, Noah Döhne, Konstantin Stadler, Sabine Grosser, Egor Byvaltcev, Anja U. Bräuer, Ulf Strauss

**Affiliations:** 1 Charité – Universitätsmedizin Berlin, corporate member of Freie Universität Berlin, Humboldt-Universität zu Berlin, and Berlin Institute of Health, Institute of Cell Biology & Neurobiology, Charitéplatz 1, 10117 Berlin, Germany; 2 Industrial Ecology Programme, NTNU - Norwegian University of Science and Technology, Trondheim, Norway; 3 Charité – Universitätsmedizin Berlin, corporate member of Freie Universität Berlin, Humboldt-Universität zu Berlin, and Berlin Institute of Health, Institute of Integrative Neuroanatomy, Berlin, Germany; 4 Research Group Anatomy, School of Medicine and Health Sciences, Carl von Ossietzky University Oldenburg, Oldenburg, Germany; 5 Research Center for Neurosensory Science, Carl von Ossietzky University Oldenburg, Oldenburg, Germany

**Keywords:** IFN, Neocortical neurons, Interferon receptor, HCN, Neuromodulation

## Abstract

**Background:**

Interferon-γ (IFN-γ, a type II IFN) is present in the central nervous system (CNS) under various conditions. Evidence is emerging that, in addition to its immunological role, IFN-γ modulates neuronal morphology, function, and development in several brain regions. Previously, we have shown that raising levels of IFN-β (a type I IFN) lead to increased neuronal excitability of neocortical layer 5 pyramidal neurons. Because of shared non-canonical signaling pathways of both cytokines, we hypothesized a similar neocortical role of acutely applied IFN-γ.

**Methods:**

We used semi-quantitative RT-PCR, immunoblotting, and immunohistochemistry to analyze neuronal expression of IFN-γ receptors and performed whole-cell patch-clamp recordings in layer 5 pyramidal neurons to investigate sub- and suprathreshold excitability, properties of hyperpolarization-activated cyclic nucleotide-gated current (*I*_h_), and inhibitory neurotransmission under the influence of acutely applied IFN-γ.

**Results:**

We show that IFN-γ receptors are present in the membrane of rat’s neocortical layer 5 pyramidal neurons. As expected from this and the putative overlap in IFN type I and II alternative signaling pathways, IFN-γ diminished *I*_h_, mirroring the effect of type I IFNs, suggesting a likewise activation of protein kinase C (PKC). In contrast, IFN-γ did neither alter subthreshold nor suprathreshold neuronal excitability, pointing to augmented inhibitory transmission by IFN-γ. Indeed, IFN-γ increased electrically evoked inhibitory postsynaptic currents (IPSCs) on neocortical layer 5 pyramidal neurons. Furthermore, amplitudes of spontaneous IPSCs and miniature IPSCs were elevated by IFN-γ, whereas their frequency remained unchanged.

**Conclusions:**

The expression of IFN-γ receptors on layer 5 neocortical pyramidal neurons together with the acute augmentation of inhibition in the neocortex by direct application of IFN-γ highlights an additional interaction between the CNS and immune system. Our results strengthen our understanding of the role of IFN-γ in neocortical neurotransmission and emphasize its impact beyond its immunological properties, particularly in the pathogenesis of neuropsychiatric disorders.

## Background

Interferons (IFN) are antiviral cytokines known for their multifaceted influence on the immune response. Based on their amino acid sequence, three types of IFNs are distinguished: IFN I to III. All of them possess specific receptors and signaling cascades [1].

IFN-γ is the only member of type II IFNs. It plays an important role in innate and adaptive immune response and induces antitumor mechanisms [2]. In addition to peripheral immune cells, central nervous system (CNS) cells such as microglia, astrocytes, dorsal root ganglion neurons, and cerebrovascular endothelial cells produce IFN-γ [3] under different conditions. Consecutively, IFN-γ concentration in the brain is elevated in certain pathologies, including multiple sclerosis [4], cerebral ischemia [5], and neurotrauma [6]. The concentration of IFNs in the brain is also elevated in cerebral and systemic viral infection [7, 8].

IFNs influence the CNS function in multiple ways [3, 9]. IFN-γ is involved in dendritic remodeling [10] and synaptic stripping by microglia [11] as well as influences the proliferation of neuronal precursor cells [12]. Dysregulation of IFN-γ production might be involved in the pathogenesis of neuropsychiatric disorders, such as depression [13] and schizophrenia [14].

The proinflammatory effects of IFN-γ are canonically mediated by binding to the multimeric IFN-γ receptor (IFN-γR), subsequently activating the Janus kinase/signal transducer and activator of transcription (JAK-STAT) pathway. Ultimately, STAT1 phosphorylation leads to the formation of STAT1-STAT1 homodimers, which regulate over 200 genes via binding of the γ-IFN activation site [15, 16]. Apart from that, IFN-γ activates alternative pathways, involving phosphatidylinositol 3-kinase (PI3-kinase) and various isozymes of protein kinase C (PKC) [17, 18]. These latter alternative pathways are also engaged in type I IFN signaling. Notably, there is evidence for activation of similar PKCs by both type I and type II IFNs in several non-neuronal cells [18, 19]. Previously, we have shown type I IFN-induced modifications of neuronal function of layer 2/3 and layer 5 pyramidal neurons that were PKC mediated. In particular, type I IFNs induced an increase in sub- and suprathreshold excitability [20, 21] due to PKC-mediated concerted action on multiple ion channels, including a decrease in hyperpolarization-activated cyclic nucleotide-gated cation (HCN) channel-mediated hyperpolarization-activated current (*I*_h_) [20, 22]. This neuromodulatory effect of type I IFNs implies a role in the altered neuronal states during inflammation and might cause some of the side effects of IFN therapy. To what extent this can be generalized to type II IFN is currently unknown. So far, information on IFN-γ influence on intrinsic neuronal properties is sparse. Synaptic effects have been reported only for long-term application and are controversial [23–25]. Nevertheless, given the overlapping signaling pathways of type I and II IFNs, we hypothesize comparable neuromodulatory effects of these IFNs. Consequently, we here establish the presence of type II IFN receptor on layer 5 pyramidal neurons in the rat neocortex and analyze the immediate consequences of IFN-γ application on neuronal excitability, ion channel function, and synaptic transmission.

## Methods

### Animals and slice preparation

Male Wistar rats obtained from Janvier labs (Saint-Berthevin, France) or from the Charité central animal facility FEM (Berlin, Germany) were kept under standard laboratory specific-pathogen-free (SPF) conditions. All procedures were performed in agreement with the European Communities Council Directive of September 22, 2010 (2010/63/EU), and carried out in accordance to state of Berlin rules (registration no. T0212/14). Immunohistochemical experiments as well as RNA extraction were done in postnatal day (P)20 and P60 rats. Acute coronal brain slices for electrophysiological recordings were prepared from P10 to P84 rats mainly according to Stadler et al. [22]. Differing from the procedure used in that publication slices were cut in 2 °C cold carbogenated (95% O_2_, 5% CO_2_), sucrose artificial cerebrospinal fluid (sACSF) containing (in mM) 85 NaCl, 2.5 KCl, 1 NaH_2_PO_4_, 7 MgCl_2_, 26 NaHCO_3_, 50 sucrose, 10 D(+)-glucose (all from Carl Roth, Karlsruhe, Germany), and 0.5 CaCl_2_ (Merck, Darmstadt, Germany). Slices were allowed to recover in 33 ± 1 °C warm sACSF for 30 min and subsequently stored at room temperature (RT) in a solution that contained (in mM) 92 NaCl, 2.5 KCl, 1.2 NaH_2_PO_4_, 30 NaHCO_3_, 25 D(+)-glucose (Carl Roth), 5 sodium ascorbate, 20 2-[4-(2-hydroxyethyl)piperazin-1-yl]ethanesulfonic acid (HEPES), 3 sodium pyruvate (Sigma-Aldrich, Steinheim, Germany), 2 thiourea (VWR Chemicals, Radnor, PA, USA), 2 MgSO_4_, and 2 CaCl_2_ (Merck).

### RNA extraction, cDNA synthesis and *ifngr1* amplification

Neocortex was harvested from three male Wistar rats and homogenized in TRIzol (Invitrogen, Carlsbad, CA, USA) reagent. Total RNA was extracted using the TRIzol reagent according to the manufacturer’s protocol (Invitrogen). The concentration and purity of the isolated total RNA were determined by spectrophotometric analysis of 5 μg total RNA using the High-Capacity cDNA Archive Kit (Applied Biosystems, Carlsbad, CA, USA) according to the manufacturer’s protocol. Control reaction was performed without MultiScribe reverse transcriptase. The quality of the amplified cDNA (with and without MultiScribe reverse transcriptase) was controlled by *β-actin* PCR. For amplification of *ifngr1* cDNA, the primers *ifngr1* 5′ (5′-aag ctt gct ctc tgt ggt aaa aaa tgc-3′) spanning bases 921–941, and *ifngr1* 3′ (5′-ctg cag tta gga cag ttc ctg ggt atc-3′), complementary to bases 1453–1473 with an amplification length from 552 bp of the *ifngr1* cDNA (GenBank accession no. NM_053783), were used. Primers for the amplification of the control gene *β-actin* were as follows: *β-actin* 5′-primer (5′-cac cac agc tga gag gga aat cgt gcg tga-3′) spanning the bases 681–710 and *β-actin* 3′-primer (5′-att tgc ggt gca cga tgg agg ggc cgg act-3′) complementary to bases 1169–1198 with an amplificate length of 520 bp for *β-actin* cDNA (GenBank accession number NM_031144.3). Due to intron inclusion, the same primers result in an amplificate length of 730 bp when genomic DNA serves as template, thus this primer combination detects possible contamination with genomic sequences.

PCR was performed in 25-μl final volume containing 1-mM deoxynucleotide triphosphates (dNTPs) (Bioline, London, UK), 2.5 units GoTaq Polymerase (Promega, Fitchburg, WI, USA), 2.5 μl 10× buffer including 2.5 M MgCl_2_ (Promega), 10 μM of each primer, and 1 μl of each cDNA for all molecules analyzed using a thermal cycler (Duo Cycler Software version 2.3.0.0, VWR, Radnor, PA, USA). For *ifngr1* and *β-actin*, the cycle program was 2 min 95 °C, 30 × [95 °C, 30 s; 63 °C, 30 s; 72 °C, 60 s], and 5 min 72 °C. The *ifngr1* fragment was cloned into the TA-TOPO vector (Invitrogen) and sequence of *ifngr1* cDNA confirmed.

### Immunoblotting

For Western blot analysis from cytosol and membrane protein, the extract of the neocortex from individual P20 or P60 rats was subjected to 10% sodium dodecyl sulfate polyacrylamide gel electrophoresis (SDS-PAGE) under reducing conditions and then blotted to a nitrocellulose membrane (Whatman, Dassel, Germany) by semi-dry protein transfer. Blots were blocked for 1 h at RT in 5% skimmed milk (Sigma-Aldrich)/0.1% Tween/1× PBS and then incubated overnight at 4 °C with a 1:1000 dilution of rabbit-anti-IFN-γ-Rα (C-20), sc-700 (Santa Cruz Biotechnology Inc., Dallas, TX, USA), and 1:10.000 dilution of mouse-anti-β-actin, AC-74 (Sigma-Aldrich). After three washes in 1× PBST, the membranes were incubated with a 1:5000 dilution of an anti-mouse Alexa Fluor 488 or 1:10.000 anti-rabbit horseradish peroxidase-labeled antibody (GE Healthcare, Chicago, IL, USA) for 1.5 h at RT. Immunoreactive bands were detected using ECL Western blotting detection reagents (GE Healthcare). As internal loading control β-actin expression was used throughout, chemiluminescence and fluorescence images were taken using the BioRad ChemiDoc MP System (Bio-Rad Laboratories, Hercules, CA, USA).

### Immunohistochemistry

Male Wistar rats were deeply anesthetized with 1.2 to 1.5 ml of a cocktail of 25 mg/ml ketamine, 1.2 mg/ml xylazine, and 0.25 mg/ml acepromazine and transcardially perfused with saline (0.9% NaCl), followed by 4% paraformaldehyde (PFA) in 0.1 M phosphate buffer (PB) (2% di-sodium hydrogen phosphate heptahydrate and 0.2% sodium di-hydrogen phosphate monohydrat (both from Carl Roth)). The brain was removed and post-fixed in the PFA/PB fixative solution overnight at 4 °C. Next, coronal sections (50 μm) of the forebrain were cut with a vibratome (Microm HM 650 V, Thermo Fisher Scientific, Waltham, MA, USA), washed in 0.1 M PB, and blocked with 0.1 M PB including 1% BSA (Serva Electrophoresis GmbH, Heidelberg, Germany) and 0.1% TritonX-100 (Sigma-Aldrich) overnight at 4 °C. Sections were subsequently incubated with anti-IFN-γ-Rα (C-20): sc-700 (1:500) (Santa Cruz Biotechnology Inc.) and anti-MAP2 (1:250) (Sigma-Aldrich) in blocking solution (0.1 M PB including 1% BSA) overnight at 4 °C. After several washes in 0.1 M PB, the sections were incubated with the second antibodies: Alexa 488-labeled goat anti-rabbit or Alexa 568-labeled goat anti-mouse (both 1:500) (Molecular Probes Inc., Eugene, OR, USA) in blocking solution for 2 h at RT. Sections were washed in 0.1 M PB before mounting. Confocal micrographs of fluorescent specimens were taken using an upright Leica TCS SL SP2 confocal microscope (Leica Microsystems, Wetzlar, Germany). Scanning was performed using an HCX PL APO CS 63x/NA1.4 oil objective at a resolution of 512 × 512 pixels in z-stack steps of 0.1 μm. Different wavelengths of fluorescence channels were imaged separately to rule out spill over. Images are presented as single scans and projections of stacks (27–52 sections). Images were processed in brightness and contrast using Fiji (https://fiji.sc/; RRID: SCR_002285, [26]).

### Interferon

For all experiments, we used Chinese hamster ovary (CHO) derived recombinant IFN-γ (U-Cytech, Utrecht, Netherlands) in a final concentration of 1000 IU ml^−1^. The lyophilized product was reconstituted in sterile double-distilled water, and small aliquots were stored at − 20 °C.

### Patch clamp recordings

Individual slices were transferred to a submerged recording chamber containing carbogenated (95% O_2_, 5% CO_2_), artificial cerebrospinal fluid (ACSF) composed of (in mM) 119 NaCl, 2.5 KCl, 1 NaH_2_PO_4_, 1.3 MgCl_2_, 26 NaHCO_3_, 10 D(+)-glucose (Carl Roth), and 2.5 CaCl_2_ (Merck).

Somatic whole-cell recordings were performed in visually identified layer 5 pyramidal neurons (mean capacitance 220.8 ± 19.7 pF, *n* = 58). The pipette solution for voltage clamp *I*_h_ recordings contained (in mM) 120 KMeSO_4_ (ICN Biomedicals, Eschwege, Germany), 20 KCl, 4 NaCl, 14 Na-phosphocreatine (Carl Roth), 0.5 ethylene glycol-bis(2-aminoethylether)-*N*,*N*,*N*′,*N*′-tetraacetic acid (EGTA), 10 HEPES, 4 Mg^2+^-ATP, and 0.3 GTP with 0.1 cAMP (Sigma-Aldrich) (pH 7.25, 288 mOsm). *I*_h_ was pharmacologically isolated [27] by modifying the ACSF (in mM, 10 KCl and NaH_2_PO_4_ omitted) and blocking confounding currents by 400 μm Ba^2+^, 1 mM Ni^2+^ (Merck), 1 μm tetrodotoxin (TTX), 10 μm 6-cyano-7-nitroquinoxaline-2,3-dione (CNQX), 25 μm D-(-)-2-amino-5-phosphonopentanoic acid (DAP-5), 10 μm bicuculline (Tocris, Bristol, UK), 5 mM 4-aminopyridine (4-AP), and 10 mM tetraethylammonium (TEA) (Sigma-Aldrich).

All inhibitory currents were recorded in the presence of 20 μM CNQX and 25 μM DAP-5 (both from Tocris) to block ionotropic glutamate receptors. Evoked inhibitory postsynaptic currents (eIPSCs) were elicited at a somatic holding potential of − 70 mV by square pulses (100 μs, 5–40 μA) every 30 s via a glass electrode filled with ACSF positioned approx. 100 μm laterally and 150 μm apically from the soma, using a stimulus isolator (ISO-Flex, Science Products, Hofheim, Germany). IFN-γ was applied when eIPSC amplitudes remained comparable for at least 10 min. Pipette solution for eIPSC and sIPSC recordings contained (in mM) 140 CsCl (Sigma-Aldrich), 4 NaCl, 1 MgCl_2_ (Carl Roth), 10 HEPES, 0.1 EGTA, 0.3 GTP, 2 Mg^2+^-ATP, and 5 QX-314 (Sigma-Aldrich) [pH 7.2, 292 mOsm]. Spontaneous IPSCs (sIPSCs) and miniature IPSCs (mIPSCs) were recorded at a holding potential of − 60 mV. For mIPSC recordings, 1 μM TTX was additionally present in the ACSF and the pipette solution contained (in mM) 130 KCl, 10 Na-phosphocreatine, 1 MgCl_2_ (Carl Roth), 1 CaCl_2_ (Merck), 11 EGTA, 10 HEPES, 2 Mg^2+^-ATP, and 0.3 GTP (Sigma-Aldrich) [pH 7.2, 290 mOsm]. IPSCs were gathered from 1 to 3 min before and after application of IFN-γ as negative deflections > 5 × RMS noise.

Electrophysiological characterization of neurons was hampered in experiments on inhibition due to the specific intracellular solutions. Therefore, biocytin (0.1%, Invitrogen) filling and post hoc staining was applied to a subset (*n* = 15) to monitor accuracy of visual selection. For morphological identification, slices were fixed in 0.4% paraformaldehyde in 0.1% PB at 4 °C for 1 h. Prior to visualization of recorded neurons, slices were washed in PB and saline-buffered PB (PBS, 0.9% NaCl) subsequently blocked in PBS containing 10% normal goat serum, 0.3% TritonX-100, and 0.05% NaN_3_ for at least 1 h. Slices were then incubated with fluorescent-conjugated streptavidin (Alexa Fluor-647, 1:1000, Invitrogen), in a PBS solution containing 3% normal goat serum, 0.1% TritonX-100, and 0.05% NaN3 for 24 h at 4 °C. Before slices were mounted for imaging, they have been washed in PBS and desalted in PB. Recorded neurons were imaged and identified using a laser scanning confocal microscope (FV1000, Olympus, Japan) and sample reconstruction was done using Fiji software package [26] and neuTube [28].

For current clamp measurements, pipette solution contained (in mM) 120 potassium gluconate, 10 KCl, 10 Na-phosphocreatine, 1 MgCl_2_ (Carl Roth), 1 CaCl_2_ (Merck), 11 EGTA, 10 HEPES, 2 Mg^2+^-ATP, and 0.3 GTP (Sigma-Aldrich) (pH 7.2, 290 mOsm). Pipettes had a tip resistance between 2 and 5 MΩ.

Data were recorded with an EPC-10 USB double amplifier (HEKA, Lambrecht, Germany), digitized (minimum of 10 kHz, after Bessel filtering at 2.5 kHz), and stored using the PatchMaster software (HEKA).

A maximal series resistance of 20 MΩ with changes < 25% during recordings was tolerated. A fast (pipette) capacitive transient (*τ* < 1.5 μs, 6–13 pF) was compensated. Input resistance was calculated with a linear fit of the current clamp generated I-V plot in close vicinity of the resting potential (± 50 pA). Intersection of the linear regression of the F-I relationship (estimated in the linear range) and abscissa roughly approximated the rheobase.

### Statistical analysis

Data was tested for normal distribution using the Shapiro-Wilk test. In case of normal distribution, paired Student’s *t* tests were used to test for significant differences. In case of significant deviation from normal distribution (*P* > 0.05), the non-parametric Wilcoxon signed-rank test was used. Kinetic analysis of the 10% biggest amplitudes of the mIPSCs was fitted by a single exponential equation (*y* = *y*0 + A1(e − *x*/τ1)), and the cumulative frequency of mIPSCs and sIPSCs was analyzed using a two-sample Kolmogorov-Smirnov test. Data are presented as mean ± standard error of the mean (SEM). Results with *P* < 0.05 were regarded as statistically significant. Statistics were performed with Origin2019 (OriginLab, Northhampton, MA, USA) or Statview v.457 (Abacus Concepts Inc., CA, USA).

## Results

### IFN-γ receptor (IFN-γR) is expressed in neocortical layer 5 pyramidal neurons

**Fig. 1:**
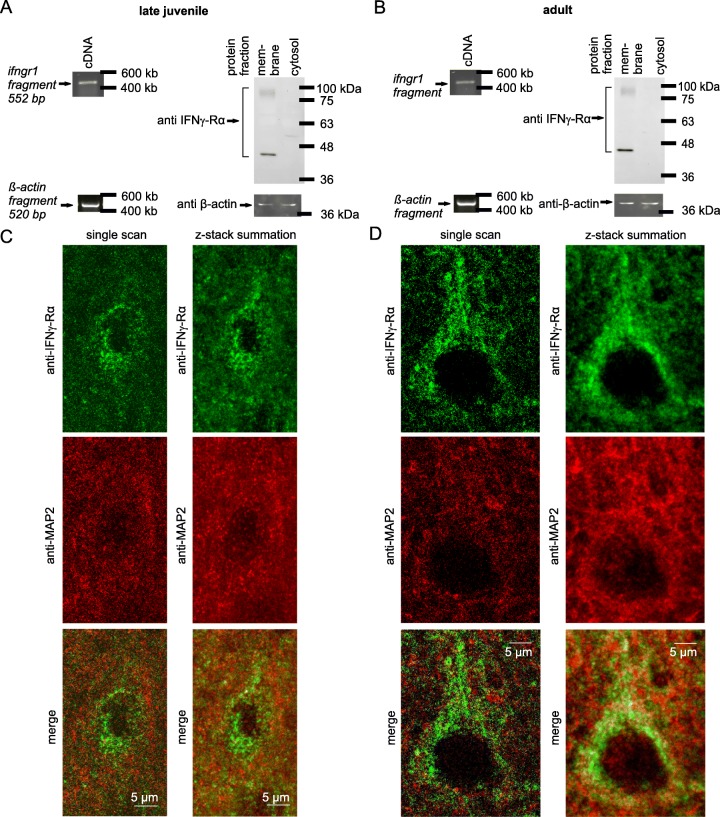
Endogenous IFNγRα is expressed in neocortical layer 5 neurons of late juvenile and adult rats. **a**, **b** Semi-quantitative RT-PCR analysis and Western blots were performed on neocortical tissue of P20 (**a**, late juvenile) and P60 rats (**b**, adult). Note that PCR analysis and Western blot were done on the same animal, i.e., neocortical tissue of one hemisphere was used for PCR, while the correspondent neocortex was used for Western blotting. The results shown are representative for three independent experiments, respectively. Left panels, semi-quantitative RT-PCR analysis of *ifngr1* mRNA, showing transcriptional gene expression of IFN-γR in the neocortex. The size of the expected fragments corresponds to the expected size (552 base pairs for *ifngr1* fragment). Loading control, *β-actin* cDNA fragments (520 base pairs). Right panels, Western blot of neocortical tissue from the same animal. Detected protein bands range from 45 to 100 kDa (96 kDa predicted by the primary structure of IFNGR1). Similar protein species were previously found to specifically bind IFN-γ [30]. Therefore, we regard the bands to be either full-length or fragmented IFN-γRα that might be post-translationally modified. Alexa 488-labeled β-actin was used as loading control. For estimation of the molecular weight, the commercial weight markers Hyperladder I (for PCR; Bioline) and BlueStar Prestained Protein Marker (for Western blot; Nippon Genetics, Tokyo, Japan) were used. **c**, **d** Examples of immunohistochemical co-staining of neocortical tissue in layer 5 with anti-IFNγ-Rα (1:500) and anti-MAP2 (neuronal marker) (1:250) in P20 (**c**) and P60 (**d**) rats. Left, single scans for accurate z-localization of Alexa 488-labeled IFNγ receptors α and Alexa 568-labeled MAP2. Right, summations of z-stacks consist of 26 (**c**) or 52 single sections (**d**)

IFN-γR expression has been confirmed for several brain regions [24, 29]. Information on the cellular localization of the receptor, however, is currently missing for many neuron types. Here we provide evidence for the expression of IFN-γR on neocortical layer 5 neurons. Using semi-quantitative RT-PCR analysis, we first assessed the transcriptional level and demonstrated that mRNA of the receptor is expressed in late juvenile and adult cortical tissue (Fig. 1a, b, left). Subsequent Western blot studies detected posttranslational expression of the ligand binding receptor α chain (IFN-γRα, also known as IFNGR1 or CD119) only in the membrane fractions derived from neocortical tissue (Fig. 1a, b, right). This suggests that interferon receptors are present on membranes of neocortical cells as astrocytes, microglia, neurons, or cerebrovascular endothelial cells. To visualize the local expression of the receptor, we used immunohistochemical staining with anti-IFN-γRα and anti-MAP2 in neocortical layer 5 of brain slices. IFN-γRα was clearly detectable in neuronal somata and weakly in dendrites, as shown by co-localization with the neuronal marker MAP2 (Fig. 1c, d). The presence of IFN-γRs in the cell membrane suggested the existence of the entire heteromeric IFN-γR at the surface of, and therefore, an influence of IFN-γ on neocortical layer 5 pyramidal neurons.

### IFN-γ application leaves neuronal excitability of layer 5 pyramidal neurons unchanged

**Fig. 2:**
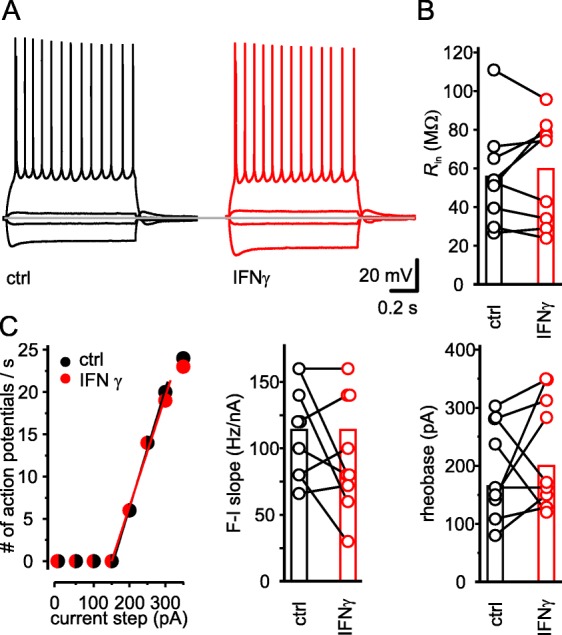
IFN-γ does not acutely alter excitability of neocortical layer 5 neurons. Direct application of 1000 IU ml^−1^ IFN-γ for 20 min did neither change suprathreshold nor subthreshold excitability. **a** Example traces of a layer 5 pyramidal neuron before (black) and upon IFN-γ application (red) showing comparable firing and subthreshold behavior. Traces resemble voltage responses to rectangular current injections of − 300, − 50, 50, and 250 pA, respectively. The dotted gray line indicates a potential of − 72 mV. **b** Input resistance, as a measure of subthreshold excitability, remained unchanged upon the application of IFN-γ. **c** Action potential frequency of an example neuron plotted as a function of the input current (left). Population data reveal that neither F-I slope (middle) nor rheobase (right) was affected by IFN-γ. As two neurons showed overlapping F-I slopes, the corresponding dots in the line series are slightly dispersed from each other. Lines represent the fit through the linear part of the curve. *R*_s_ remained constant (*R*_s-ctrl_ = 7.6 ± 0.6 MΩ vs. *R*_s-IFN-γ_ = 7.3 ± 0.5 MΩ, *n* = 9; *P* = 0.23, paired *t* test) throughout the experiments. The average age of animals used for these current clamp experiments was P30 ± 2.8. In this and all following figures, columns indicate mean and open circles in line series resemble individual values before (black) and after (red) application of IFN-γ; *P < 0.05, **P < 0.01, ***P < 0.001

Crosstalk for type I and type II IFNs has not only been shown for the canonical pathways [31], but also for the activation of PKC [15, 19]. We have shown previously that type I IFNs boost suprathreshold excitability via activation of PKC [21]. To elucidate whether IFN-γ similarly triggers a change in excitability, we investigated the firing behavior of layer 5 pyramidal neurons before and 20 min after direct application of IFN-γ. In contrast to type I IFNs that augment the suprathreshold excitability of about 65% of layer 2/3 and layer 5 pyramidal neurons [20], IFN-γ did not change the suprathreshold excitability in layer 5 pyramidal neurons as reflected in similar F-I slope (ctrl 114 ± 11 Hz/nA vs. IFN-γ 96 ± 14 Hz/nA; *n* = 9; *P* = 0.23, paired *t* test) and rheobase, i.e., the minimal current amplitude for action potential induction (ctrl 194 ± 27.7 pA vs. IFN-γ 225 ± 32 pA; *n* = 9, *P* = 0.40, paired *t* test) (Fig. 2a, c). The resting membrane potential (*V*_m-ctrl_ = − 68 ± 0.1 mV vs. *V*_m-IFN-γ_ = − 67 ± 0.2 mV; *n* = 9, *P* = 0.16, Wilcoxon signed-rank test) remained constant during the experiments. Furthermore, the input resistance (*R*_in_) as a measure of subthreshold excitability also remained unchanged upon IFN-γ application (*R*_in-ctrl_ 55.7 ± 0.8 MΩ vs. *R*_in-IFN-γ_ 59.7 ± 0.9 MΩ; *n* = 9, *P* = 0.46, paired *t* test; Fig. 2b). This precludes the possibility that we might have recorded mainly from neurons that were marginally or not affected in their suprathreshold response. In summary, the lack of effect of IFN-γ on sub- and suprathreshold excitability indicates either a failed crosstalk or additional interfering mechanisms.

### IFN-γ reduces *I*_h_ amplitude and slows its kinetics

Given the well-established PKC activation by IFN-γ [17, 18], the lack of a neuromodulatory effect might be due to a failed PKC-mediated channel modulation upon IFN-γ. As we have robust information on the effects of type I IFNs on *I*_h_ [22] mediated by the PKC pathway [21], we next analyzed the influence of IFN-γ on *I*_h_.

**Fig. 3:**
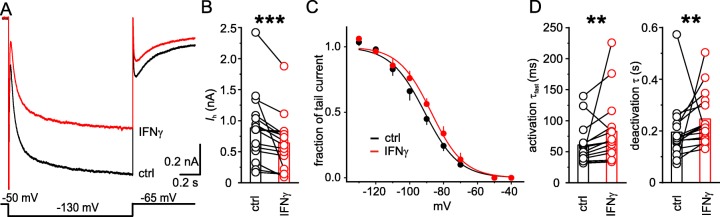
*I*_h_ is inhibited by IFN-γ in neocortical layer 5 neurons. **a** After 10 min, 1000 IU ml^−1^ IFN-γ decreased maximum amplitude of *I*_h_ and slowed its activation and deactivation as exemplified by traces of pharmacologically isolated and offline leak subtracted *I*_h_ before (black) and upon (red) direct IFN-γ application. *I*_h_ was elicited by the voltage step shown at the bottom. **b** IFN-γ reduced the *I*_h_ amplitude in all investigated neurons. **c** For the graph steady-state activation, curves were constructed from average relative tail currents (SEM given as bars) upon returning to − 65 mV from *I*_h_ activation, plotted against preceding activating voltages before (black) and after the application of IFN-γ (red). Fits of Boltzmann function are superimposed. Note that *V*_1/2_ after IFN-γ is depolarized and cannot explain the decrease in maximum *I*_h_. **d** Application of IFN-γ decelerated the fast component of activation (quantified by an increase of *τ*_fast_, left) and *I*_h_ deactivation as indicated by increased deactivation times (right). *R*_s_ remained constant (*R*_s-ctrl_ 9.7 ± 0.7 MΩ vs. *R*_s-IFN-γ_ 10.0 ± 0.7 MΩ, *n* = 16; *P* = 0.3, paired *t* test) throughout the experiments. Average age of animals for this series of experiments P35.6 ± 11.0

Acute application of IFN-γ (1000 IU ml^−1^) led to a reduction of *I*_h_ amplitude by about 25% from *I*_h-ctrl_ = 881.4 ± 136.8 pA to *I*_h-IFN-γ_ = 640.7 ± 110.2 pA (*n* = 16; *P* < 0.001, Wilcoxon signed-rank test). This attenuation occurred in all investigated neurons within 10 min of application (Fig. 3a, b). The reduction of *I*_h_ amplitude was associated with a depolarization of the midpoint of activation *V*_1/2_ (Fig. 3c; *V*_1/2-ctrl_ = − 89.0 ± 1.0 mV to *V*_1/2-IFN-γ_ = − 86.2 ± 1.2 mV; *n* = 16, *P* < 0.05, paired *t* test). This shift in *V*_1/2_ cannot account for the reduction of the peak amplitude and argues against a classical rundown. In addition, we estimated *I*_h_ amplitudes at full activation under both conditions.

In our previous study, we observed a distinct effect of type I IFNs on *I*_h_ kinetics [22]. The same effect appeared upon application of IFN-γ: the current onset was faster, i.e., the fast time constant *τ*_fast_ increased from *τ*_fast-ctrl_ = 60.9 ± 8.3 ms to *τ*_fast-IFN-γ_ = 82.8 ± 13.9 ms (*n* = 16; *P* < 0.01, Wilcoxon signed-rank test, Fig. 3d), whereas the slow time constant *τ*_slow_ remained unchanged (*τ*_slow-ctrl_ = 560.4 ± 122.7 ms vs. *τ*_slow-IFN-γ_ = 1096.0 ± 403.8 ms; *n* = 16, *P* = 0.27, Wilcoxon signed-rank test). Besides the activation kinetics, also the deactivation slowed down (*τ*_ctrl_ = 194.8 ms ± 28.7 ms to *τ*_IFN-γ_ = 246.0 ms ± 25.7 ms; *n* = 16, *P* < 0.01, Wilcoxon signed-rank test, Fig. 3d). The modulation of *I*_h_ by IFN-γ corresponds qualitatively and quantitatively to previously observed effects of type I IFNs on *I*_h_ [22] that were due to activation of PKC [21]. These data establish a link between IFN-γ, PKC activation, and attenuated *I*_h_, rendering a concerted action of IFN-γ on several ionic channels likely.

### Inhibitory synaptic transmission is increased in amplitude by elevated IFN-γ levels

These conflicting results—the lack of change in neuronal excitability on one, the overlap between IFN-γ and type I IFNs regarding channel modulation on the other hand—prompted us to consider other putative IFN-γ targets. Taking the role of neuronal inhibition to restrict neocortical excitability [32–34] into account, we hypothesized that IFN-γ augments GABAergic inhibition.

**Fig. 4:**
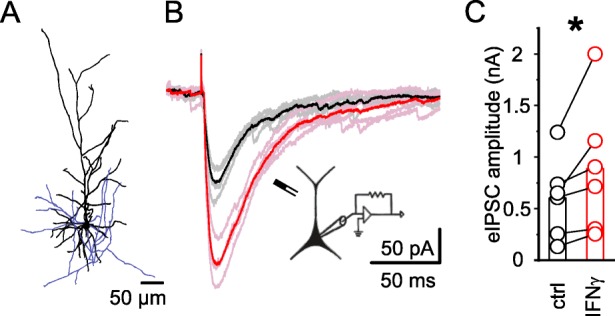
IFN-γ increases the amplitude of evoked IPSCs in neocortical layer 5 pyramidal neurons. Electrically evoked IPSCs were recorded in the presence of CNQX and DAP5 to block ionotropic glutamate receptors. **a** Exemplary layer 5 pyramidal neuron stained with biocytin and reconstructed to ensure neuronal identity when electrophysiological characterization was impaired by recording conditions. Soma and dendrites are represented in black, the axon in blue. **b** Example traces of IPSCs evoked before (gray) and after 30 min of IFNγ application (1000 IU ml^−1^; rose). Averaged traces are shown in black (before IFN-γ application) and red (after IFN-γ application). A scheme of the experimental setup, including the apicolaterally positioned bipolar stimulation electrode, is depicted below. **c** Maximum amplitudes of all eIPSCs increased under the influence of IFN-γ. Analysis of eIPSC amplitudes was performed before and 20 to 40 min after the application of IFN-γ. Holding current (507.9 ± 189.0 pA vs. 1285.9 ± 626.0 pA, *n* = 6; *P* = 0.22, Wilcoxon signed-rank test) and *R*_s_ (*R*_s-ctrl_ = 9.2 ± 2.1 MΩ vs. *R*_s-IFN-γ_ = 9.4 ± 2.2 MΩ, *n* = 6; *P* = 0.7, Wilcoxon signed-rank test) remained comparable. Average rat age P23.7 ± 0.8

To investigate a possible impact of IFN-γ on GABAergic transmission, we evoked IPSCs (eIPSCs), before and 20 to 40 min after application of IFN-γ using electrical stimulation. IFN-γ increased the maximum amplitude of eIPSCs in the presence of glutamate receptor blockers by 48% (ctrl 605 ± 160 pA to IFN-γ 889 ± 263 pA; *n* = 6, *P* < 0.05, Wilcoxon signed-rank test; Fig. 4b, c). The area under the curve (AUC) that represents the total membrane charge transfer showed a trend towards increase (ctrl 3.33 ± 1.89 nA vs. IFN-γ 4.40 ± 2.48 nA; *n* = 6, *P* = 0.21, Wilcoxon signed-rank test), maybe due to bulk-induced increased uptake.

**Fig. 5:**
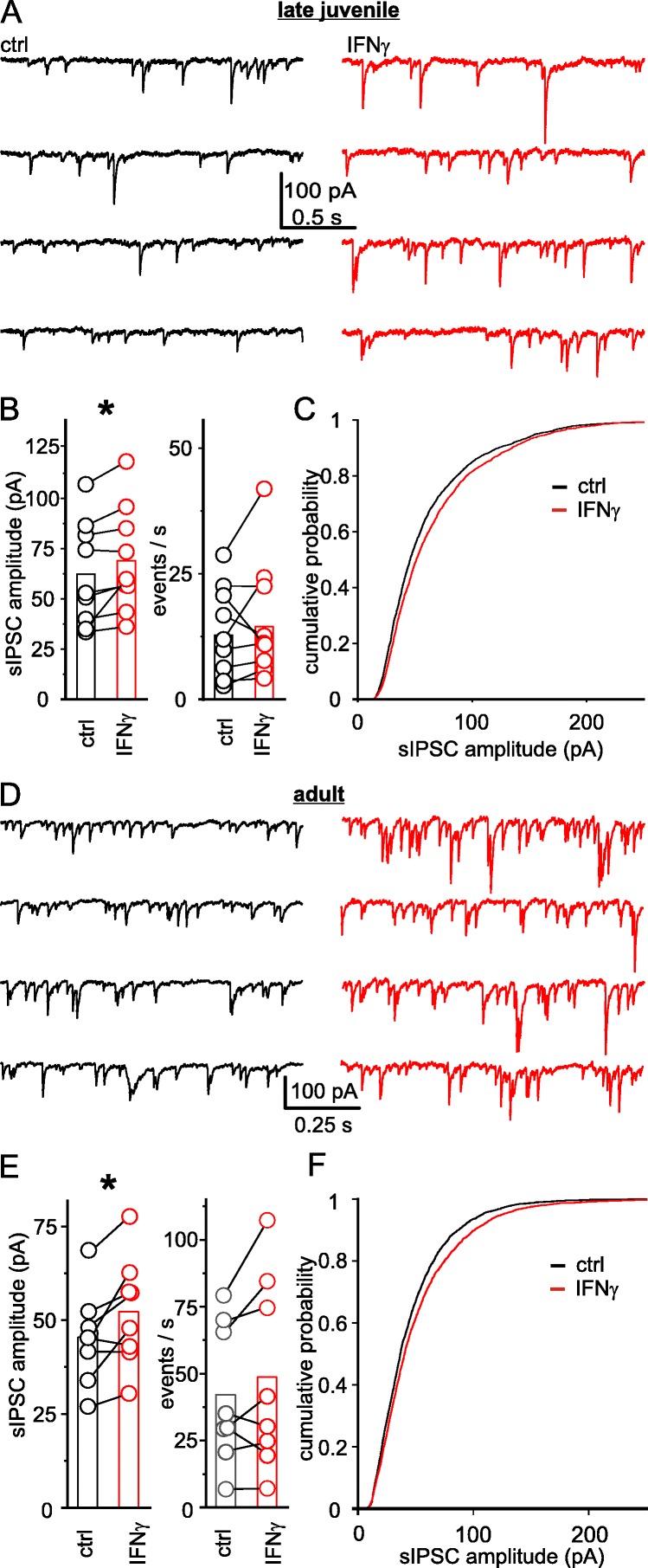
IFN-γ increases the amplitude of spontaneous IPSCs in neocortical layer 5 pyramidal neurons of late juvenile and adult rats. Spontaneous IPSCs were recorded in the presence of CNQX and DAP5 to block ionotropic glutamate receptors. **a**, **d** Example traces of sIPSCs of late juvenile (**a**) and adult (**d**) rats before (black) and after application of IFN-γ (1000 IU ml^−1^; 20 min; red). **b**, **e** IFN-γ application (20 to 30 min) increased sIPSC amplitudes of pyramidal neurons of late juvenile (**b**) and adult (**e**) rats (left) but left sIPSC frequency (right) unchanged. **c**, **f** Cumulative sIPSC amplitudes in neurons of late juvenile (**c**) and adult (**f**) rats after application of IFN-γ (red) were shifted to bigger amplitudes when compared with control (black). Holding current (for late juvenile rats 325.7 ± 91.9 pA vs. 412.5 ± 72.5 pA, *n* = 9; *P* = 0.13, Wilcoxon signed-rank test/for adult rats 135.5 ± 28.2 pA vs. 135.0 ± 19.5 pA, *n* = 8; *P* = 0.97, paired *t* test) and *R*_s_ (for late juvenile rats *R*_s-ctrl_ = 9.2 ± 2.1 MΩ vs. *R*_s-IFN-γ_ = 9.4 ± 2.2 MΩ, *n* = 9; *P* = 0.7, Wilcoxon signed-rank test/for adult rats *R*_s-ctrl_ = 7.1 ± 1.0 MΩ vs. *R*_s-IFN-γ_ = 7.0 ± 0.5 MΩ, *n* = 8; *P* = 0.8, paired *t* test) remained comparable. Mean age of animals P19.0 ± 1.0 (late juvenile) and P60.0 ± 0.6 (adult)

To address GABAergic transmission on the level of single synapses, we measured spontaneous IPSCs (sIPSCs) 20 to 30 min after application of IFN-γ. The amplitude of sIPSCs increased by 14.6% (from ctrl 62.4 ± 8.7 pA to IFN-γ 69.5 ± 8.8 pA; *n* = 9, *P* < 0.05, paired *t* test) while the frequency of sIPSCs remained unchanged (ctrl 13.7 ± 3.0 events s^−1^ vs. IFN-γ 15.7 ± 4.0 events s^−1^; *n* = 9, *P* = 0.42, paired *t* test, Fig. 5a–c). Comparison of the largest (≥ 60 pA) sIPSCs that are putatively action potential driven showed comparable amplitudes (ctrl 99.0 ± 7.8 pA vs. IFN-γ 100.9 ± 6.8 pA; *n* = 9, *P* = 0.16, paired *t* test) or frequencies (ctrl 4.0 ± 1.1 events s^−1^ vs. IFN-γ 5.8 ± 1.3 events s^−1^; *n* = 9, *P* = 0.2, paired *t* test).

Most electrophysiological recordings so far were carried out on late juvenile rats, on average aged around P20. Because we cannot exclude that developmental changes may interfere with effects of IFN-γ, we firstly used linear regression analysis on age and effect size for all electrophysiological experiments but found no correlation (probability; Pearson correlation coefficient): input resistance (*p* 0.69; *r* 0.15), rheobase (0.64; − 0.18), F-I slope (0.99; − 0.002), hyperpolarization-induced current amplitude (0.11; − 0.41), evoked IPSC amplitude (0.39; 0.44), spontaneous IPSC amplitude (0.92; 0.03), and miniature IPSC amplitude (0.22; 0.43). Secondly, because the inhibitory circuitry develops rather slow, rendering GABAergic transmission development susceptible to extrinsic influences [35], we investigated whether the synaptic effect of IFN-γ persists in adult rats. In detail, we recorded sIPSCs at P60 and found—similar to juvenile rats—an amplitude increase of sIPSCs by 15.7% (from ctrl 45.4 ± 4.4 pA to IFN-γ 52.2 ± 5.2 pA; *n* = 8, *P* < 0.05, paired *t* test) while frequencies remained comparable (ctrl 42.1 ± 9.2 events s^−1^ vs. IFN-γ 48.7 ± 12.6 events s^−1^; *n* = 8, *P* = 0.18, paired *t* test, Fig. 5d–f). Here, the amplitude of the largest (≥ 60 pA) sIPSCs increased by 11.8% (from ctrl 88.8 ± 3.3 pA to IFN-γ 99.3 ± 5.3 pA; *n* = 8, *P* < 0.05, paired *t* test) whereas their frequency remained unchanged (ctrl 12.1 ± 4.3 events s^−1^ vs. IFN-γ 18.4 ± 7.1 events s^−1^; *n* = 8, *P* = 0.07, paired *t* test).

**Fig. 6:**
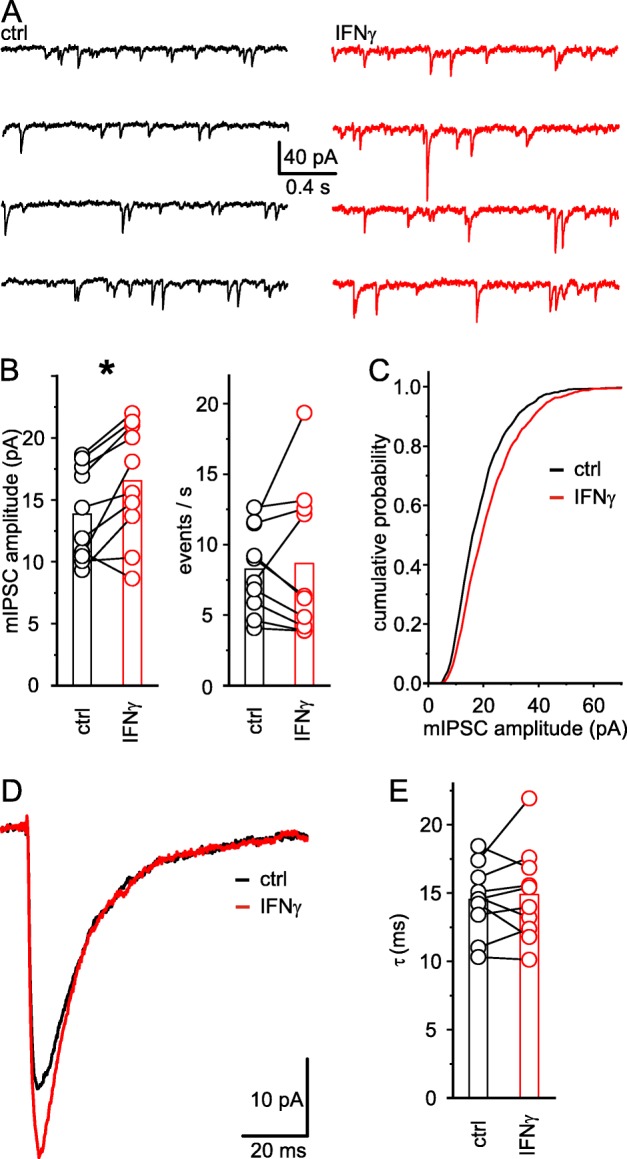
IFN-γ increases mIPSC amplitudes in neocortical layer 5 neurons. **a** Example traces of mIPSCs recorded in the presence of CNQX, DAP5, and TTX before (black) and upon application of IFN-γ (1000 IU ml^−1^; 20 min; red). **b** Mean mIPSC amplitudes (left) increased upon application of IFN-γ, while mIPSC frequency (right) remained unaltered. **c** Cumulative relative frequency graph of mIPSC amplitudes for an example neuron shows an increase in amplitudes after application of IFN-γ (red) in comparison to the initial state (black). **d** Example trace of a single mIPSC before (black) and upon application of IFN-γ (red). **e** IFN-γ did not influence mIPSC decay time. *R*_s_ remained constant (*R*_s-ctrl_ 14.1 ± 0.7 MΩ vs. *R*_s-IFN-γ_ 14.5 ± 0.8 MΩ; *n* = 10; *P* = 0.55, paired *t* test) throughout the experiments. Average rat age P16.1 ± 1.9

Finally, we recorded miniature IPSCs (mIPSCs) in the presence of glutamate receptor blockers and TTX to analyze the effects of IFN-γ on inhibitory transmission, while excluding any action potential driven responses. After 20 min of application, IFN-γ increased the amplitude of mIPSCs by 20% from ctrl 13.8 ± 1.2 pA to IFN-γ 16.5 ± 1.5 pA (*n* = 10; *P* < 0.01, paired *t* test) without any effect on the frequency (ctrl 8.2 ± 1.0 events s^−1^ vs. IFN-γ 8.7 ± 1.7 events s^−1^; *n* = 10, *P* = 0.85, Wilcoxon signed-rank test; Fig. 6a–c). Kinetic analysis of the 10% biggest amplitudes indicated no changes of the decay time (Fig. 6d, e).

These results provide evidence for an acute augmentation of GABAergic transmission by IFN-γ.

## Discussion

Our main finding is that short-term IFN-γ application provokes complex changes in neuronal function of neocortical layer 5 pyramidal neurons, enabled by the presence of type II IFN receptors on these neurons. In detail, IFN-γ, when acutely released, augments inhibitory currents in neocortical layer 5 pyramidal neurons. Augmented perisomatic inhibition links our seemingly conflicting other findings: on the one hand, the decrease and slowing of *I*_h_ mimics the effect we previously found for type I IFNs [22], which indicates a comparable, PKC-mediated mechanism [21]. On the other hand, the therefore expected increase in neuronal sub- and suprathreshold excitability (as seen in the majority of neurons upon type I IFNs [20]) was entirely absent.

Our newly found short-term effects of IFN-γ on GABAergic responses in neocortical pyramidal neurons in acute slice preparations add to the knowledge on long-term IFN-γ effects on inhibition. In line with our findings of an augmented inhibition, layer 1 interneurons respond to meningeal IFN-γ release and increase tonic inhibition in layer 2 projection neurons [36]. Further support of our findings comes from other brain regions such as the hippocampus, where IFN-γ augmented synaptic inhibition as shown in the brain slices of late juvenile rats by an increase in frequency of spontaneous and miniature IPSCs [37] or in culture at peak synaptogenesis by increased spontaneous IPSC frequency and miniature IPSC amplitude [23], both at later time points, i.e., at hours to days after IFN-γ exposure. The latter authors linked their findings to increased neuronal activity, an interaction that recently has been deciphered [38]. Such a mechanism is not likely to account for our findings because we blocked glutamatergic transmission with CNQX for AMPA and DAP-5 for NMDA receptors in our experiments. However, in contrast to these rather supporting findings on IFN-γ-induced augmentation, there is also evidence for an attenuation of inhibition. IFN-γ reduced or even depolarized GABAergic activity in neurons in the dorsal horn of the spinal cord [39] and increased IFN-γ levels diminished inhibition by reducing frequency but not amplitude of spontaneous and miniature inhibitory postsynaptic current (IPSC) [40] in hippocampal CA1 neurons. Adding another layer of complexity, the IFN-γ-induced reduction in GABA release has been shown to increase hippocampal excitability [40, 41], and IFN-γ may induce increase in hippocampal excitatory synaptic activity and AMPA receptor clustering without obvious changes in GABAergic transmission [25]. These inconsistent findings in diverse regions of the central nervous system certainly demand further studies of localized and age-dependent neuronal IFN-γ effects.

Although the similarity of the effects of IFN-γ and type I IFNs on *I*_h_ suggests a comparable mechanism of acute action, i.e., PKC mediation, the obvious differences in influencing neuronal excitability together with the diverse effects of mere IFN-γ application in different preparations and brain regions described previously provoke several speculations. The most parsimonious hypotheses are (1) subcellular receptor location: IFN-γ receptors may preferentially interact with inhibitory GABA_A_ receptors at the soma of pyramidal neurons whereas type I IFN receptors might rather modulate dendritic conductances. A partial overlap may explain the lack of suprathreshold effects in a small subset (14/39) of IFN-β treated pyramidal neurons [20]. (2) Different subcellular PKC subtype or intracellular PKC target distribution [42]. (3) Differential action on resident interneuron subclasses, depending on their ion channel composition. (4) IFNs as cytokines rarely act alone; thus, effects should be considered in conjunction with other cytokines present or even induced in the local environment. For instance, IFN-γ may evoke either TNFα production in glial cells that, in turn, could influence (excitatory) synaptic function [43] or CX3CL1 [44, 45]. Interestingly, the latter mimics the here described IFN-γ effect, i.e., it augmented sIPSC amplitudes but not frequencies when acutely applied [46]. However, this effect was specific for serotonergic neurons, and CX3CL1 induction by IFN-γ was not described to be in the time frame of the effects we observe, yet. (5) Finally, although our (specifically due to the time course) and several other studies are in favor of a direct neuromodulatory effect of cytokines, we cannot entirely exclude the possibility that a variable (micro-)glial influence affects short- or long-term neuromodulation [47], in particular, because IFN-γ induced CX3CL1 release [44, 45] that might have an impact on GABAergic networks, at least in development [48].

Our experiments suggest that early enhancement of GABAergic inhibition may protect against pro-excitatory events, as previously proposed by [36]. However, further studies are needed here because the significance of the results beyond the level of the brain slice still needs to be clarified. Pro- and anti-excitatory incidents might be simultaneously induced by IFN-γ as HCN1 reduction has been shown to be sufficiently counteracted by GABAergic enhancement [34]. Dampening neuronal activity might even counteract the morphological changes indicative for upcoming degeneration as dendritic beads initiated by Ca^2+^ permeable receptor complexes of IFN-γR and GluR1 [24] or be beneficial before IFN-γ forces neurons to instruct inhibitory synapse loss in viral *déjà vu* disease [11]. Although our experiments in acute brain slices do not provide data on chronic impact, initial augmentation of GABAergic inhibition might lead to a rearrangement that finally results in the chronic outcomes described by others (see above). Whatever the exact mechanisms are, our finding might show the onset of the suggested neuroprotective role. This would be in line with findings that GABAergic activity was enhanced in surviving neurons in primary cortical cultures following rabies or influenza A infection [49, 50].

Recent research points to a role of IFN-γ (and therewith putatively changes in inhibition) in the pathophysiology of other diseases besides viral infections. IFN-γ was reported to be involved in depressive behavior in humans [51], and intraventricular delivery of IFN-γ caused a depressive and anxiety-like behavior in mice due to dysfunction of the cannabinoid receptor CB1Rs that (if functioning) reduces GABA transmission in the striatum [52]. Our findings on an altered synaptic transmission by IFN-γ add a new aspect on the role of pro-inflammatory cytokines in epilepsy. So far, IL-1β, TNFα, and IL-6 are regarded as major pro-epileptic players [42]. In contrast, IFN-γ-induced augmentation of inhibition might rather constrain seizures, although its absence in development seems to diminish seizures later in life [53].

## Conclusion

The data improve our understanding of the role of IFN-γ as a neuromodulatory cytokine [3] and reveal potential mechanisms of altered behavior and perception during early states of infectious diseases. Our data once more emphasize that immune and nervous system do act autonomously but mutually affect each other’s function. Knowledge on IFN-γ-induced neuromodulation is important because it is of clinical relevance, due to IFN-γ presence in the CNS under pathological conditions and even beyond, since it might influence social behavior [36].

## Data Availability

The datasets used and/or analyzed during the current study are available from the corresponding author on reasonable request.

## Abbreviations

4-AP: 4-Aminopyridine
ACSF: Artificial cerebrospinal fluid
ATP: Adenosine triphosphate
BSA: Bovine serum albumin
cAMP: Cyclic adenosine monophosphate
cGMP: Cyclic guanosine monophosphate
CHO: Chinese hamster ovary
CNQX: 6-Cyano-7-nitroquinoxaline-2,3-dione
CNS: Central nervous system
DAP-5: D-(-)-2-amino-5-phosphonopentanoic acid
EGTA: Ethylene glycol-bis(2-aminoethylether)-*N*,*N*,*N*′,*N′*-tetraacetic acid
HCN: Hyperpolarization activated cyclic nucleotide gated
HEPES: 2-[4-(2-Hydroxyethyl)piperazin-1-yl]ethanesulfonic acid
IFN: Interferon
*I*_h_: Hyperpolarization activated cyclic nucleotide gated nonselective cationic current
IPSC: Inhibitory postsynaptic current (e - evoked, s - spontaneous, m- miniature)
JAK: Janus kinase
P: Postnatal day
PB: Phosphate buffer
PBS: Saline-buffered phosphate buffer
PCR: Polymerase chain reaction
PKC: Protein kinase C
RT: Room temperature
SDS-PAGE: Sodium dodecyl sulfate polyacrylamide gel electrophoresis
STAT: Signal transducer and activator of transcription
TEA: Tetraethylammonium
TTX: Tetrodotoxin

## References

[1] Owens T, Khorooshi R, Wlodarczyk A, Asgari N (2014). Interferons in the central nervous system: a few instruments play many tunes. Glia..

[2] Schroder K, Hertzog PJ, Ravasi T, Hume DA (2004). Interferon-gamma: an overview of signals, mechanisms and functions. J Leukoc Biol.

[3] Monteiro S, Roque S, Marques F, Correia-Neves M, Cerqueira JJ (2017). Brain interference: revisiting the role of IFNγ in the central nervous system. Prog Neurobiol.

[4] Traugott U, Lebon P (1988). Interferon-gamma and Ia antigen are present on astrocytes in active chronic multiple sclerosis lesions. J Neurol Sci.

[5] Li HL, Kostulas N, Huang YM, Xiao BG, van der Meide P, Kostulas V (2001). IL-17 and IFN-gamma mRNA expression is increased in the brain and systemically after permanent middle cerebral artery occlusion in the rat. J Neuroimmunol.

[6] Lau LT, Yu AC (2001). Astrocytes produce and release interleukin-1, interleukin-6, tumor necrosis factor alpha and interferon-gamma following traumatic and metabolic injury. J Neurotrauma.

[7] Heremans H, Billiau A, De Somer P (1980). Interferon in experimental viral infections in mice: tissue interferon levels resulting from the virus infection and from exogenous interferon therapy. Infect Immun.

[8] Frei K, Leist TP, Meager A, Gallo P, Leppert D, Zinkernagel RM (1988). Production of B cell stimulatory factor-2 and interferon gamma in the central nervous system during viral meningitis and encephalitis. Evaluation in a murine model infection and in patients. J Exp Med.

[9] Reyes-Vázquez C, Prieto-Gómez B, Dafny N (2012). Interferon modulates central nervous system function. Brain Res.

[10] Monteiro S, Ferreira FM, Pinto V, Roque S, Morais M, de Sá-Calçada D (2016). Absence of IFNγ promotes hippocampal plasticity and enhances cognitive performance. Transl Psychiatry.

[11] Di Liberto G, Pantelyushin S, Kreutzfeldt M, Page N, Musardo S, Coras R (2018). Neurons under T cell attack coordinate phagocyte-mediated synaptic stripping. Cell.

[12] Sun L, Tian Z, Wang J (2010). A direct cross-talk between interferon-gamma and sonic hedgehog signaling that leads to the proliferation of neuronal precursor cells. Brain Behav Immun.

[13] Schmidt FM, Lichtblau N, Minkwitz J, Chittka T, Thormann J, Kirkby KC (2014). Cytokine levels in depressed and non-depressed subjects, and masking effects of obesity. J Psychiatr Res.

[14] Arolt V, Rothermundt M, Wandinger KP, Kirchner H (2000). Decreased in vitro production of interferon-gamma and interleukin-2 in whole blood of patients with schizophrenia during treatment. Mol Psychiatry.

[15] Platanias LC (2005). Mechanisms of type-I- and type-II-interferon-mediated signalling. Nat Rev Immunol.

[16] Boehm U, Klamp T, Groot M, Howard JC (1997). Cellular responses to interferon-gamma. Annu Rev Immunol.

[17] Deb DK, Sassano A, Lekmine F, Majchrzak B, Verma A, Kambhampati S (2003). Activation of protein kinase C delta by IFN-gamma. J Immunol.

[18] Srivastava KK, Batra S, Sassano A, Li Y, Majchrzak B, Kiyokawa H (2004). Engagement of protein kinase C-theta in interferon signaling in T-cells. J Biol Chem.

[19] Hald A, Andrés RM, Salskov-Iversen ML, Kjellerup RB, Iversen L, Johansen C (2013). STAT1 expression and activation is increased in lesional psoriatic skin. Br J Dermatol.

[20] Hadjilambreva G, Mix E, Rolfs A, Müller J, Strauss U (2005). Neuromodulation by a cytokine: interferon-beta differentially augments neocortical neuronal activity and excitability. J Neurophysiol.

[21] Reetz O, Stadler K, Strauss U (2014). Protein kinase C activation mediates interferon-β-induced neuronal excitability changes in neocortical pyramidal neurons. J Neuroinflammation.

[22] Stadler K, Bierwirth C, Stoenica L, Battefeld A, Reetz O, Mix E (2014). Elevation in type I interferons inhibits HCN1 and slows cortical neuronal oscillations. Cereb Cortex.

[23] Brask J, Kristensson K, Hill RH (2004). Exposure to interferon-gamma during synaptogenesis increases inhibitory activity after a latent period in cultured rat hippocampal neurons. Eur J Neurosci.

[24] Mizuno T, Zhang G, Takeuchi H, Kawanokuchi J, Wang J, Sonobe Y (2008). Interferon-gamma directly induces neurotoxicity through a neuron specific, calcium-permeable complex of IFN-gamma receptor and AMPA GluR1 receptor. FASEB J.

[25] Vikman KS, Owe-Larsson B, Brask J, Kristensson KS, Hill RH (2001). Interferon-gamma-induced changes in synaptic activity and AMPA receptor clustering in hippocampal cultures. Brain Res.

[26] Schindelin J, Arganda-Carreras I, Frise E, Kaynig V, Longair M, Pietzsch T (2012). Fiji: an open-source platform for biological-image analysis. Nat Methods.

[27] Strauss U, Kole MH, Bräuer AUA, Pahnke J, Bajorat R, Rolfs A (2004). An impaired neocortical Ih is associated with enhanced excitability and absence epilepsy. Eur J Neurosci.

[28] Feng Linqing, Zhao Ting, Kim Jinhyun (2015). neuTube 1.0: A New Design for Efficient Neuron Reconstruction Software Based on the SWC Format. eneuro.

[29] Robertson B, Kong G, Peng Z, Bentivoglio M, Kristensson K (2000). Interferon-gamma-responsive neuronal sites in the normal rat brain: receptor protein distribution and cell activation revealed by Fos induction. Brain Res Bull.

[30] Aguet M, Dembić Z, Merlin G (1988). Molecular cloning and expression of the human interferon-gamma receptor. Cell..

[31] Gough DJ, Messina NL, Hii L, Gould JA, Sabapathy K, Robertson APS (2010). Functional crosstalk between type I and II interferon through the regulated expression of STAT1. PLoS Biol.

[32] Fellous JM, Rudolph M, Destexhe A, Sejnowski TJ (2003). Synaptic background noise controls the input/output characteristics of single cells in an in vitro model of in vivo activity. Neuroscience..

[33] Chance FS, Abbott LF, Reyes AD (2002). Gain modulation from background synaptic input. Neuron..

[34] Chen X, Shu S, Schwartz LC, Sun C, Kapur J, Bayliss DA (2010). Homeostatic regulation of synaptic excitability: tonic GABA(A) receptor currents replace I(h) in cortical pyramidal neurons of HCN1 knock-out mice. J Neurosci.

[35] Micheva KD, Beaulieu C (1997). Development and plasticity of the inhibitory neocortical circuitry with an emphasis on the rodent barrel field cortex: a review. Can J Physiol Pharmacol.

[36] Filiano AJ, Xu Y, Tustison NJ, Marsh RL, Baker W, Smirnov I (2016). Unexpected role of interferon-γ in regulating neuronal connectivity and social behaviour. Nature.

[37] Flood L, Korol SV, Ekselius L, Birnir B, Jin Z (2019). Interferon-γ potentiates GABAA receptor-mediated inhibitory currents in rat hippocampal CA1 pyramidal neurons. J Neuroimmunol.

[38] Flores CE, Nikonenko I, Mendez P, Fritschy J-M, Tyagarajan SK, Muller D (2015). Activity-dependent inhibitory synapse remodeling through gephyrin phosphorylation. Proc Natl Acad Sci U S A.

[39] Vikman KS, Duggan AW, Siddall PJ (2007). Interferon-gamma induced disruption of GABAergic inhibition in the spinal dorsal horn in vivo. Pain.

[40] Zhu PJ, Huang W, Kalikulov D, Yoo JW, Placzek AN, Stoica L (2011). Suppression of PKR promotes network excitability and enhanced cognition by interferon-γ-mediated disinhibition. Cell.

[41] Müller M, Fontana A, Zbinden G, Gähwiler BH (1993). Effects of interferons and hydrogen peroxide on CA3 pyramidal cells in rat hippocampal slice cultures. Brain Res.

[42] Vezzani A, Viviani B (2015). Neuromodulatory properties of inflammatory cytokines and their impact on neuronal excitability. Neuropharmacology.

[43] Beattie EC, Stellwagen D, Morishita W, Bresnahan JC, Ha BK, von Zastrow M (2002). Control of synaptic strength by glial TNFalpha. Science.

[44] Imaizumi T, Matsumiya T, Fujimoto K, Okamoto K, Cui X, Ohtaki U (2000). Interferon-gamma stimulates the expression of CX3CL1/fractalkine in cultured human endothelial cells. Tohoku J Exp Med.

[45] Yoshida H, Imaizumi T, Fujimoto K, Matsuo N, Kimura K, Cui X (2001). Synergistic stimulation, by tumor necrosis factor-alpha and interferon-gamma, of fractalkine expression in human astrocytes. Neurosci Lett.

[46] Heinisch S, Kirby LG (2009). Fractalkine/CX3CL1 enhances GABA synaptic activity at serotonin neurons in the rat dorsal raphe nucleus. Neuroscience.

[47] Ta Thuy-Truc, Dikmen Hasan Onur, Schilling Simone, Chausse Bruno, Lewen Andrea, Hollnagel Jan-Oliver, Kann Oliver (2019). Priming of microglia with IFN-γ slows neuronal gamma oscillations in situ. Proceedings of the National Academy of Sciences.

[48] Bertot C, Groc L, Avignone E (2019). Role of CX3CR1 signaling on the maturation of GABAergic transmission and neuronal network activity in the neonate hippocampus. Neuroscience.

[49] Ladogana A, Bouzamondo E, Pocchiari M, Tsiang H (1994). Modification of tritiated gamma-amino-n-butyric acid transport in rabies virus-infected primary cortical cultures. J Gen Virol.

[50] Brask J, Owe-Larsson B, Hill RH, Kristensson K (2001). Changes in calcium currents and GABAergic spontaneous activity in cultured rat hippocampal neurons after a neurotropic influenza A virus infection. Brain Res Bull.

[51] Schmidt FM, Schröder T, Kirkby KC, Sander C, Suslow T, Holdt LM (2016). Pro- and anti-inflammatory cytokines, but not CRP, are inversely correlated with severity and symptoms of major depression. Psychiatry Res.

[52] Mandolesi G, Bullitta S, Fresegna D, Gentile A, De Vito F, Dolcetti E (2017). Interferon-γ causes mood abnormalities by altering cannabinoid CB1 receptor function in the mouse striatum. Neurobiol Dis.

[53] Getts DR, Matsumoto I, Müller M, Getts MT, Radford J, Shrestha B (2007). Role of IFN-gamma in an experimental murine model of West Nile virus-induced seizures. J Neurochem.

